# Implementation of a Structured Preclinical Simulation Tool for Locator Housing Pick-Up Training

**DOI:** 10.3390/dj14050285

**Published:** 2026-05-11

**Authors:** Po-Hsu Chen, Chin-Chuan Fu, Daniel A. Givan

**Affiliations:** Department of Restorative Sciences, University of Alabama at Birmingham School of Dentistry, Birmingham, AL 35233, USA; ccfu@uab.edu (C.-C.F.); dgivan@uab.edu (D.A.G.)

**Keywords:** implant overdenture, Locator attachment, preclinical simulation

## Abstract

**Background/Objectives:** Delivering consistent preclinical instruction for implant attachment procedures can be challenging in large dental cohorts. This report describes the development and implementation of institutionally produced training tools designed to support Locator housing pick-up exercises for second-year predoctoral dental students. **Methods:** Modified typodont-based simulation tools were integrated into the preclinical curriculum. Clear dentures and gypsum models were fabricated to allow visualization of seating relationships and identification of common interferences. Complete seating of the denture was verified using inspection windows, flange evaluation, and polyvinylsiloxane disclosing materials before housings were incorporated with autopolymerizing acrylic resin. After each session, components were collected, inspected, and prepared for reuse in subsequent cycles. Learner perceptions were obtained through an anonymous voluntary survey. **Results:** The configuration enabled visualization of seating conditions and identification of misalignment during the exercise. Removal of anterior teeth reduced material use and emphasized posterior stabilization during the pick-up procedure. Of 83 learners, 28 completed the survey (34% response rate), with responses tending toward agreement across items (mean range: 4.5–4.9/5), indicating favorable learner perceptions of the exercise and its organization within the scheduled laboratory period. Across three academic cycles, six dentures required replacement, whereas all gypsum models remained serviceable and no additional fabrication was necessary. **Conclusions:** This structured simulation approach provided an alternative method for delivering Locator housing pick-up training in a high-volume preclinical environment. The model allowed repeated implementation of the exercise across academic cycles.

## 1. Introduction

Mandibular two-implant-retained complete overdenture has been considered the standard of care for edentulous patients [1,2]. Two implants placed on the anterior region of the fully edentulous mandible with attachments benefit the retention and stability of the denture; the improved chewing function, nutritional status, and speech by implant-retained overdentures contribute to patients’ satisfaction compared to conventional mandibular complete dentures without using implants [3]. The Locator Attachment System (Zest Dental Solutions, Carlsbad, CA, USA) is widely used for this purpose due to its higher tolerance of implant divergence compared to other stud attachment systems [4], and the lower requirement of restorative space and better cleanability compared to the bar attachments [5]. Moreover, the Locator Attachment System supports a variety of implant connections [6], simplifying the learning curve of manipulating the attachments while restoring different brands of implants.

The direct or indirect techniques might be applied to pick up the metal housings of the Locator Attachment by dentures. The direct technique refers to picking up the housings intraorally; in contrast, the indirect technique requires the impression along with corresponding Locator copings to record the relationship of abutments and edentulous ridge, and the pick-up process is accomplished during the denture processing stage [7]. Ahmed [8] and Ibrahim et al. [9] compared two techniques on mandibular overdentures, and found no significant differences regarding the retention of dentures. However, Elsewedy et al. discovered that the direct technique presented higher retention than the indirect technique when measured one week after the pick-up procedure [10]. While the indirect technique reduces chair time and avoids direct tissue contact with the acrylic monomer, the distortion from the process of implant location transferring and denture processing might cause incomplete seating of the denture on the tissue. The direct technique was considered more accurate by overcoming the limitations above, contributing to decreased need for prosthetic aftercare [11], which was adopted as the method practiced in the present study; however, it is technique-sensitive in controlling the prosthesis position intraorally during the curing of the autopolymerizing resin and preventing acrylic resin from entering undercut areas of attachments [12].

Dental providers who apply the Locator Attachment System in patient care must be proficient in performing housing pick-up procedures without compromising denture seating on the edentulous ridge. To support the development of this skill, simulation-based learning provides a controlled, low-risk environment for procedural training and has been incorporated into multiple areas of dental education, including fixed prosthodontics [13], removable dentures [14,15], and implant dentistry [16]. These applications often require adaptation to institutional needs, particularly in high-volume predoctoral training settings. While the implant manufacturer provided training tools for practicing the direct pick-up procedure with resin models and gingiva-colored denture bases, the limited number of tools did not support the regular dental school class size, which led to the need for frequent recycling during practice sessions, hindering equal learning access for all students. Additionally, the manufacturer’s inconsistent availability of tools and shipping disruptions affected course scheduling, prompting the need for institution-developed training tools with modified designs to better support the educational mission.

## 2. Materials and Methods

The target learners were 83 second-year dental students enrolled in a preclinical complete denture course. The implant-retained overdenture module was introduced following conventional complete denture lectures and simulation exercises. Prior to a 20-min laboratory orientation session, students received 3.5 h of didactic instruction covering the fundamental principles of implant-retained and implant-supported removable prostheses. Step-by-step instructional materials were provided through a learning management system (Canvas, Instructure, Salt Lake City, UT, USA) and were accessible to both instructors and learners during and after the course. Students were organized into groups of six to seven, with three groups assigned to each two-hour session, ensuring that no more than 21 students participated at one time. Four sessions were conducted to provide hands-on experience for the entire cohort. Instruction was delivered by two prosthodontic faculty members and three prosthodontic residents serving as module leaders.

**Figure 1:**
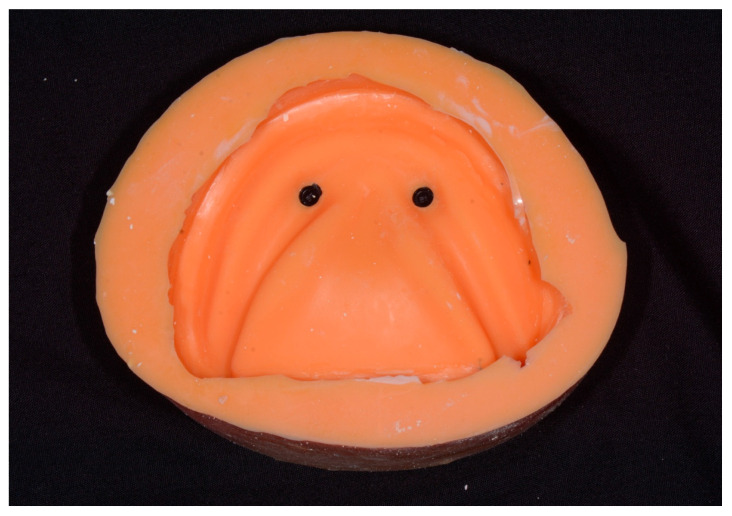
The silicone mold with impression copings embedded.

An existing preclinical edentulous model was duplicated using silicone duplicating material (Elite Double 22 Fast, Zhermack, Badia Polesine, Italy) and type IV gypsum (ResinRock, Whip Mix, Louisville, KY, USA) to establish a prototype. Two Locator analogs were embedded in positions appropriate for a mandibular two-implant overdenture. With impression copings in place, the duplicating material was used to create molds for definitive casts (Figure 1), which were poured in gypsum with embedded Locator analogs.

**Figure 2:**
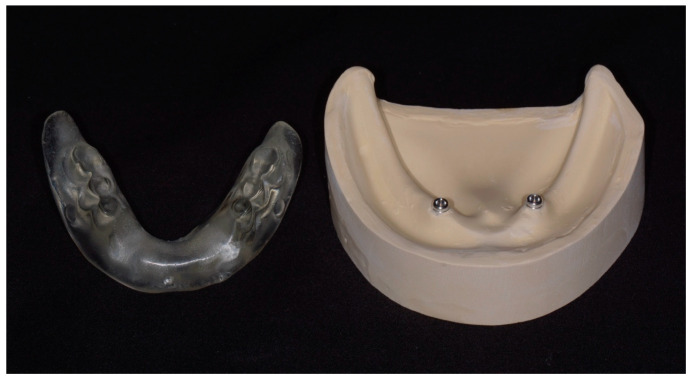
Modified simulation tool for Locator housing pick-up training, consisting of a gypsum model and a clear denture.

A prototype overdenture was derived from a complete denture by scanning with a desktop scanner (E4, 3Shape, Copenhagen, Denmark) and fabricating with a 3D printer (Pro S, SprintRay, Los Angeles, CA, USA) using a clear light-curable resin (Surgical Guide 3, SprintRay, Los Angeles, CA, USA). The anterior teeth were intentionally removed to enhance visualization during the pick-up procedure. Polyvinylsiloxane impression material (Aquasil Ultra+ LV, Dentsply Sirona, Charlotte, NC, USA) was applied to the tissue surface to improve adaptation to the model. The modified denture was rescanned, and inspection windows were digitally created beneath the buccal cervical areas of the posterior teeth. Definitive overdentures for each learner were produced using the same resin. A total of 100 clear dentures and 25 gypsum models were fabricated to accommodate the class size, with additional units prepared to allow for potential procedural complications (Figure 2).

Students practiced attachment pick-up according to the manufacturer’s instructions [7]. Each step was guided by module leaders, and learners received feedback before proceeding. The educational objectives of this simulation exercise were to enable students to (1) achieve complete seating of the overdenture on the edentulous ridge, (2) accurately perform Locator attachment housing pick-up using autopolymerizing resin, and (3) recognize and manage common procedural errors, such as misalignment of dentures and incomplete housing pick-up.

**Figure 3:**
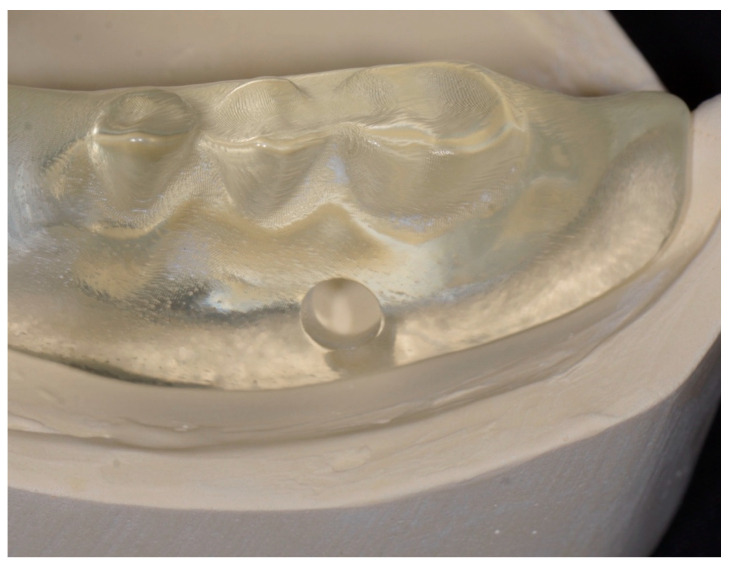
Inspection windows, adaptation of the denture flanges to the model, and the use of clear resin facilitated verification of complete seating.

**Figure 4:**
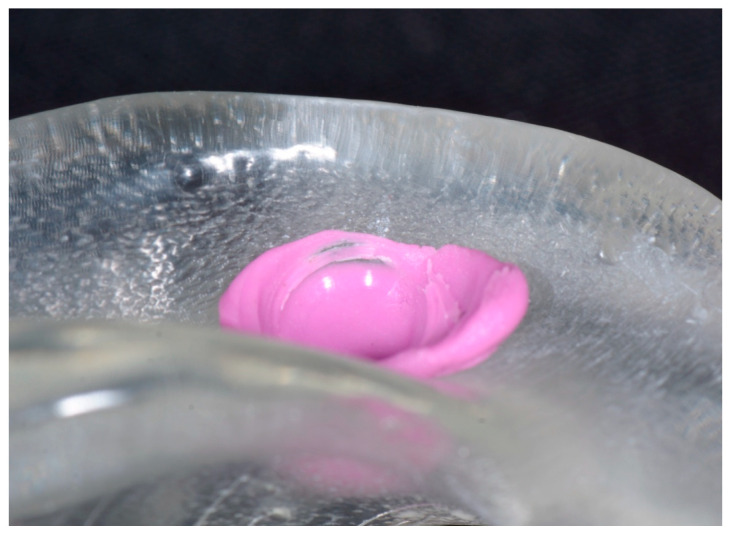
Students used polyvinylsiloxane bite registration material to identify interference between the housings and the denture base.

**Figure 5:**
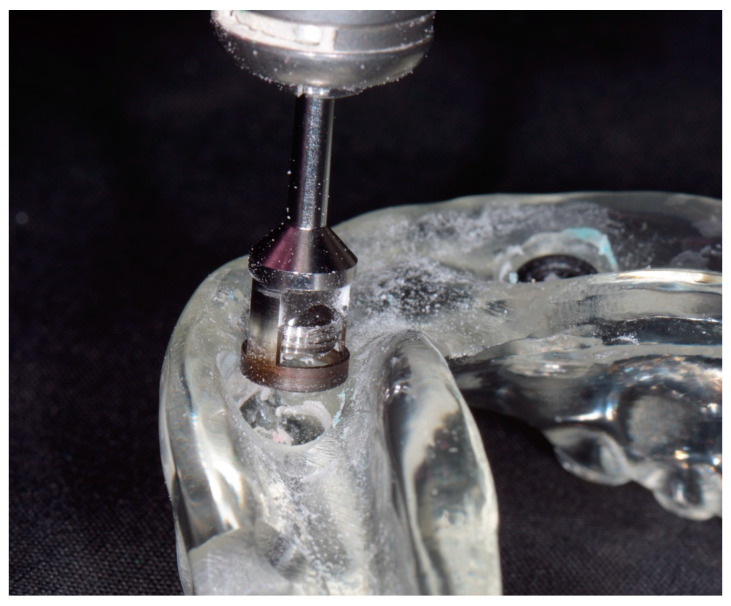
A Trephine Bur was used to remove the embedded metal housings while preserving denture integrity, permitting reuse in subsequent sessions.

1. Students positioned block-out spacers and housings on both Locator analogs. Instructors verified proper positioning.
2. Students relieved the recessed areas of the dentures using rotary instrumentation (H71.11.050 HP TC Round Carbide, Brasseler, Savannah, GA, USA) on the tissue surface to accommodate the housings and ensure full seating on the models. Seating was verified through inspection windows and denture flanges (Figure 3). Polyvinylsiloxane bite registration material (Regisil Rigid, Dentsply Sirona, Charlotte, NC, USA) was used to identify interferences (Figure 4), which were adjusted accordingly.

3. After confirming full seating, students used clear autopolymerizing acrylic resin (Ortho-Jet, Lang Dental, Wheeling, IL, USA) to pick up the housings using the bead-brush technique, in which monomer was first placed into the recess area, followed by incremental addition of polymer powder to build the resin matrix [17,18]. Gypsum models were protected with petroleum jelly (Vaseline, Unilever, Englewood Cliffs, NJ, USA). A water bath (Digital Water Bath, Whip Mix, Louisville, KY, USA) was used, with the entire model-denture assembly immersed in 122 °F (50 °C) water, to accelerate the polymerization of the acrylic resin. Repeat attempts were guided as needed.
4. Instructors assessed completion by demonstrating the use of the Locator Core Tool (Zest Dental Solutions, Carlsbad, CA, USA) for removal and insertion of nylon inserts. Completion was recorded on a pass/fail basis for course credit. A pass was defined as completion of the project within the two-hour session, whereas a fail required remediation.
5. After completing the exercise, students returned all materials to the laboratory coordinator.
6. For subsequent sessions, previously used gypsum models were inspected to confirm the absence of acrylic resin overflow and reused. New dentures were distributed.
7. For subsequent academic cycles, housings were removed using a trephine bur (Zest Dental Solutions, Carlsbad, CA, USA), and minor resin additions were made to facilitate repeated practice (Figure 5).

An anonymous, voluntary online survey of the Spring 2024 cohort was administered following the laboratory sessions using Qualtrics XM (Qualtrics, Provo, UT, USA). Participation had no impact on academic standing, and completion of the questionnaire was considered implied consent. The survey assessed learners’ agreement with statements regarding the training tools using a 5-point Likert scale ranging from 1 (strongly disagree) to 5 (strongly agree). Free-text responses were also collected. Additionally, the reuse and durability of dentures and gypsum models were documented across the 2024–2026 cycles.

## 3. Results

### 3.1. Course Outcomes

All learners completed the laboratory exercise within the scheduled two-hour session. The preformed recesses in the dentures were intentionally smaller than the metal housings, requiring enlargement of the intaglio surface to obtain passive seating. Polyvinylsiloxane materials were applied within the recesses to identify potential interference between the dentures and housings, consistent with common clinical verification approaches. Pick-up procedures were initiated only after seating had been confirmed by the instructors.

Common challenges included insufficient application of autopolymerizing resin and removal of the denture prior to complete polymerization. These situations were managed through supervised repetition and the introduction of a water bath to facilitate more predictable curing. Difficulty in seating the printed dentures was occasionally related to undercuts in the retromylohyoid region of the original model. This was addressed through selective modification of the gypsum casts using rotary instrumentation to narrow the lingual aspect of the posterior ridge. Following this adjustment, seating was more readily achieved in subsequent cohorts.

### 3.2. Learner Perception

Survey items and aggregate responses are presented in Table 1. Twenty-eight of 83 learners participated, yielding a response rate of 34%. Responses tended toward agreement across all items, with mean scores ranging from 4.5 to 4.9 on the 5-point scale. The highest ratings were observed for clarity of project goals and adequacy of session time, whereas slightly lower ratings were noted for the ease of performing the housing pick-up. Free-text responses identified strengths, including the small-group environment, availability of instructors, visibility of component assembly, and opportunities for hands-on participation in implant attachment procedures. Feedback identifying areas for improvement was used to guide changes in later sessions, including the introduction of the water bath and the elimination of undercut over the retromylohyoid fossa areas of the gypsum model (Table 2).

### 3.3. Tools Recycling Outcomes

During the 2024 implementation, three of the 83 dentures were accidentally dropped and fractured; each was immediately replaced with a spare. All 25 gypsum models were returned intact, with no damage affecting future use. Several block-out spacers were lost and subsequently restocked, and worn or distorted nylon inserts and metal housings were replaced after the session. In 2025, no dentures or gypsum models were damaged, and no remanufacturing was required. In 2026, three dentures were damaged; however, the existing inventory remained sufficient, and additional fabrication was not necessary.

## 4. Discussion

**Figure 6:**
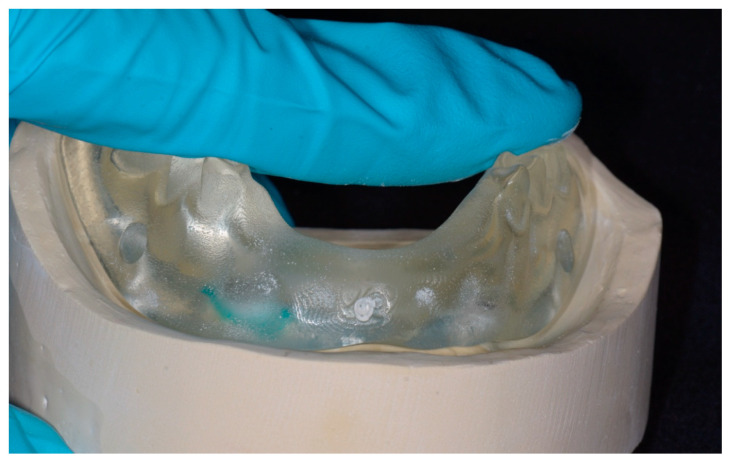
The design of the training tools supported the recommended practice of applying pressure to the posterior dentition during the direct pick-up procedure.

The present tools were designed to provide students with opportunities to practice the direct pick-up technique. The design focused on early exposure to the procedural sequence and visual cues associated with denture seating. The use of transparent materials and removal of anterior teeth allowed learners to observe the relationship between adjustments and seating, which may facilitate conceptual understanding of how procedural steps influence outcomes. In addition, the design reinforced posterior pressure during denture seating, which has been suggested to improve denture adaptation to the underlying tissue (Figure 6) [19]. The design also reduced material usage during fabrication and made common errors, such as incomplete seating and misalignment, more readily observable. The tools provided consistent access for all students without the need for repeated redistribution during the course. In-house additive manufacturing allowed efficient reproduction of the tools as needed. While annual remaking of transparent dentures may enhance the student learning experience and is technically feasible with in-house production, the decision to reuse the tools was driven by cost-efficiency considerations. The autopolymerizing acrylic resin demonstrated adequate bonding to the printed denture material, facilitating the pick-up procedure. However, bonding between the acrylic resin and printed resin models was problematic, making separation difficult and potentially limiting repeated use. This led to the adoption of gypsum models as a more practical alternative.

Various disclosing media have been recommended to verify complete denture seating, including Pressure Indicating Paste (Keystone, Gibbstown, NJ, USA) [19,20], articulating paper, occlusal spray, bite registration materials [21], and polyvinylsiloxane materials such as Fit Checker (GC America Inc., Alsip, IL, USA) [21,22,23]. In this study, bite registration materials were used to simulate clinical detection of interferences between metal housings and the intaglio surface of the denture. The use of transparent dentures with inspection windows allowed learners to visualize these interferences and observe changes following adjustments, which may support discussion of procedural rationale. Learner feedback suggested that participants perceived the exercise as helpful for clarifying procedural steps and reinforcing concepts introduced during didactic instruction.

Several limitations should be acknowledged. The simulation environment does not replicate intraoral conditions, including limited visibility, tissue resiliency, and patient-related variability. Therefore, additional supervised clinical experience is necessary for skill transfer to patient care. The use of a water bath did not fully replicate routine clinical procedures, and clarification was provided to minimize potential misunderstandings among students. The mandibular edentulous models used in this study often included undercuts in the retromylohyoid region, requiring additional adjustments during denture fabrication. The laboratory exercise did not include selection of abutment cuff height, as this would require multiple Locator abutments and increase material costs. The dentures were also susceptible to fracture if dropped, indicating a need for material optimization. Finally, comparison with previous training methods within the same cohort was not feasible due to inconsistent availability of prior tools. As a result, all students used the newly developed tools during the study period. The voluntary nature of the survey likely contributed to the relatively low participation rate, potentially limiting the representativeness of the findings and introducing a positive response bias in learner perceptions; furthermore, learner satisfaction may not accurately reflect actual learning outcomes and requires further validation. Future work may include progression toward less visible and more clinically realistic simulation conditions, such as the use of opaque materials or integration into a head simulator, to better mimic clinical settings.

## 5. Conclusions

The modified Locator pick-up training tools allowed for the delivery of a structured preclinical experience for learners within a high-volume instructional setting and may be transferable to other institutions with access to basic laboratory fabrication resources. Survey responses suggested that students were generally able to follow the procedural sequence and engage with key steps of the exercise. The approach may provide a practical framework for organizing implant attachment training within the preclinical curriculum, particularly in settings requiring standardized instruction across large groups of learners. Given that the evaluation was limited to learner perception, future studies incorporating objective performance measures may help to further clarify the educational impact of the tools.

## Figures and Tables

**Table 1:** Descriptive results of the student survey administered after the 2024 training sessions (N = 28).

	Survey Questions	Mean Scores ± Standard Deviation *	Score Range
1	I found it easy to navigate the goals of this project by following the slides and module leaders’ instructions.	4.9 ± 0.4	4–5
2	I found the pick-up of metal housings easy to achieve on the typodonts.	4.5 ± 0.7	2–5
3	I felt I had enough time to complete the project.	4.9 ± 0.3	4–5
4	I was overall satisfied with the Locator metal housings pick-up practice on the typodonts.	4.7 ± 0.6	3–5
5	Please provide any comments or feedback on the tools that were not covered by the previous questions.	N/A	N/A

* Under a 5-point Likert scale ranging from 1 (strongly disagree) to 5 (strongly agree).

**Table 2:** Free-text comments from the 2024 student survey and the corrective actions implemented in response.

	Comments	Actions
1	I was the first group to attend the lab session, and we had some issues with the metal housing pick-up because the acrylic was not drying quickly enough for us to be able to pick up both housings.	Water bath was introduced after the first group.
2	My typodont did not have a great fit, but overall I think the lab was very useful and should be kept.	The elimination of undercut over the retromylohyoid fossa areas of the gypsum model helped improve the fit.
3	I believe it helped me understand the slides by executing the project in the lab. I felt that it was a good usage of our time and I really appreciated this.	
4	I liked that it was in small groups, so we had enough faculty to help us without having to wait forever.	
5	As someone interested in implants, this provided a great service of showing me how Locators will work.	
6	I think that this procedure will go much more smoothly in the clinic now that we have had a chance to practice. It also helped solidify what we learned in the lecture a lot.	

## Data Availability

The original contributions presented in this study are included in the article. Further inquiries can be directed to the corresponding author.
